# Tropical Plant Extracts as Potential Antihyperglycemic Agents

**DOI:** 10.3390/molecules17055915

**Published:** 2012-05-18

**Authors:** Thamilvaani Manaharan, Uma Devi Palanisamy, Cheng Hwee Ming

**Affiliations:** 1 Department of Physiology, Faculty of Medicine, University Malaya, Kuala Lumpur 50603, Malaysia; 2 School of Medicine and Health Sciences, Monash University, Sunway Campus, Bandar Sunway, Selangor 46150, Malaysia

**Keywords:** type 2 diabetes mellitus, α-glucosidase, α-amylase, antioxidant, total phenolic content

## Abstract

Preliminary investigations on 14 plant extracts (obtained by ethanolic and aqueous extraction) identified those having high antioxidant and a significant total phenolic content. Antihyperglycemic, α-amylase and α-glucosidase inhibition activities were also observed. A correlation between the antihyperglycemic activity, total phenolic content and antioxidant (DPPH scavenging) activity was established. To further substantiate these findings, the possibility of tannins binding non-specifically to enzymes and thus contributing to the antihyperglycemic activity was also investigated. Our study clearly indicated that the antihyperglycemic activity observed in the plant extracts was indeed not due to non-specific tannin absorption.

## 1. Introduction

Diabetes mellitus is a metabolic disease characterized by hyperglycemia resulting from defects in insulin action, insulin secretion or both. The most common type of diabetes mellitus is type 2 diabetes mellitus, which accounts for 85 to 95% of all cases and constitutes a major and growing public health problem [1]. Type 2 diabetes mellitus is generally managed through a stepwise program of intensive therapy that consists of lifestyle and sequential addition of oral antihyperglycemic agents (OHAs) and insulin as necessary. One therapeutic approach to decrease postprandial hyperglycemia in diabetic state is by retarding absorption of glucose through inhibition of carbohydrate hydrolyzing enzymes, like α-glucosidase and α-amylase, in the digestive tract [1,2]. These drugs however risk inducing hypoglycemia and over time, lose their efficacy, have prominent side effects and fail to significantly alter the course of diabetic complications.

Plants have been suggested as a rich, source of potentially useful antidiabetic drugs [3]. Recent scientific investigations have confirmed the efficacy of many of these preparations, some of which are remarkably effective [4,5]. Although there is widespread use of herbal dietary supplements that are believed to benefit type 2 diabetes mellitus, but still there is a great interest in the development of new drugs to prevent type 2 diabetes mellitus with minimal or without any side effects and to evaluate natural products in experimental studies [5,6,7]. The objective of this study was to investigate a number of tropical plant extracts for their antioxidant ability (DPPH radical scavenging activity), antihyperglycemic activity (as α-glucosidase and α-amylase inhibitors) and as well as determine the total phenolic content. We also studied the correlation between antioxidant ability and antihyperglycemic, α-glucosidase and α-amylase inhibition activity.

## 2. Results and Discussion

### 2.1. Antihyperglycemic and Antioxidant Ability of Tropical Plant Extracts

The details of the selected tropical plants used in this study are presented in Table 1. The antihyperglycemic activity, antioxidant activity and total phenolic content of selected tropical plants extracted under both ethanolic and aqueous conditions were studied and results are as shown in Table 2 and Table 3.

**Table 1:** The selected tropical plants; scientific name, parts, local name and family.

Scientific Name	Parts	Local Name	Family
*Azadirachta indica*	Leaf	Neem	Meliaceae
*Artocarpus champeden*	Leaf	Cempedak	Moraceae
*Garcinia mangostana*	Rind	Mangosteen	Clusiaceae
*Nephelium lappaceum*	Rind and leaf	Rambutan	Sapindaceae
*Peltophorum pterocarpum*	Leaf, bark, pod and flower	Yellow flame tree	Fabaceae
*Syzygium aqueum*	Leaf	Water jambu, water apple	Myrtaceae
*Syzygium cumini*	Leaf, bark and seed	Java plum, jambolana	Myrtaceae
*Vitis vinifera*	Seed	Grape	Vitaceae

Generally, it was observed that the ethanolic extracts displayed far higher antihyperglycemic, antioxidant activity and total phenolic content than the aqueous ones. This trend was also observed in the percentage of yield of extraction in almost all the extracts. The plant *Peltophorum pterocarpum*, particularly its bark and flower parts, was seen to exhibit extremely high α-glucosidase, α-amylase, antioxidant as well as total phenolic content. This was followed by the extracts from *Syzygium cumini*, *Nephelium lappaceum*, *Syzygium aqueum* and *Garcinia mangostana.* Interestingly, it was observed that these extracts displayed activities comparable and at times higher than the commercial antihyperglycemic drug acarbose, and commercial grape seed extract.

**Table 2:** 1/EC_50_ of α-glucosidase, α-amylase and DPPH activity, TPC and yield of ethanolic extraction of various plant extracts.

Plant Extracts	Part	Ethanol				
		1/EC_50_ (mg/mL)			TPC,GAE (mg/g)	% Yield of Extraction
		α-Glucosidase	α-Amylase	DPPH		
*Azadirachta indica*	Leaf	39.8 ± 13.2	2.7 ± 0.9	1.2 ± 0.2	53.6 ± 12.5	14.4 ± 13.2
*Artocarpus champeden*	Leaf	91.7 ± 10.4	ND *	7.1 ± 1.1	556.2 ± 28.2	27.8 ± 9.6
*Garcinia mangostana*	Rind	200.0 ± 28.2	41.7 ± 3.2	11.9 ± 4.1	600.0 ± 34.9	22.0 ± 7.5
*Nephelium lappaceum*	Rind	370.4 ± 31.6	14.1 ± 8.5	15.7 ± 5.3	809.6 ± 53.2	33.2 ± 16.3
*Nephelium lappaceum*	Leaf	400.0 ± 45.7	31.2 ± 9.4	8.8 ± 1.2	321.0 ± 21.5	18.8 ± 7.3
*Peltophorum pterocarpum*	Leaf	292.3 ± 17.5	65.4 ±10.1	5.7 ± 13.2	256.2 ± 35.7	15.4 ± 2.4
*Peltophorum pterocarpum*	Bark	1,666.7 ± 596.4	400.0 ± 43.2	32.1 ± 18.6	797.6 ± 383.2	20.0 ± 11.2
*Peltophorum pterocarpum*	Pod	1,111.1 ± 857.3	49.3 ± 21.9	12.6 ± 10.2	372.6 ± 193.7	15.8 ± 7.5
*Peltophorum pterocarpum*	Flower	2,500.0 ± 966.8	123.5 ± 93.2	20.1 ± 17.3	594.0 ± 213.1	17.6 ± 12.4
*Syzygium aqueum*	Leaf	344.8 ± 175.3	23.6 ± 13.2	8.4 ± 5.6	265.4 ± 178.9	14.0 ± 12.5
*Syzygium cumini*	Leaf	769.2 ± 323.7	45.9 ± 28.2	10.9 ± 1.2	371.0 ± 197.6	1.0 ± 0.5
*Syzygium cumini*	Bark	400.0 ± 245.8	21.0 ± 17.3	6.6 ± 3.8	303.4 ± 153.8	2.8 ± 1.6
*Syzygium cumini*	Seed	625.0 ± 254.7	9.2 ± 75.8	9.1 ± 8.2	375.4 ± 268.1	8.0 ± 5.7
*Vitis vinifera* (Grape)	Seed	357.1 ±265.9	227.3 ± 145.9	34.8 ± 29.1	834.8 ± 475.5	2.6 ± 1.2
Acarbose		0.286 ± 0.08	83.3 ± 25.9			

* ND, Not detected.

It is noteworthy that *Syzygium cumini* has been extensively used in the empirical treatment of diabetes mellitus [8,9,10]. In this study, we observed that *Peltophorum pterocarpum* (bark, pod and flower) displayed far better α-glucosidase and α-amylase inhibition activity compared to the *Syzygium cumini* (leaf, bark and seed). Recently, *Garcinia mangostana* (mangosteen) rind juice has been marketed as a healthy beverage under the trade name of XANGO and it has been reported possessing several biological activities, in particular high blood pressure and blood glucose level reducing effects [11]. In this study, *Peltophorum pterocarpum* (leaf, bark, pod and flower), *Nephelium lappaceum* (rind and leaf) and *Syzygium aqueum* (leaf) showed prominent antioxidant ability and far better α-glucosidase and α-amylase inhibition activity compared to *Garcinia mangostana* (rind). It is therefore seemed essential that further studies to identify bioactive compounds and carry out *in vivo* studies involving these plants are required.

**Table 3:** 1/EC_50_ of α-glucosidase, α-amylase and DPPH activity, TPC and yield of aqueous extraction of various plant extracts.

Plant Extracts	Part	Aqueous				
		1/EC_50_ (mg/mL)			TPC GAE(mg/g)	% Yield of Extraction
		α-Glucosidase	α-Amylase	DPPH		
*Azadirachta indica*	Leaf	3.8 ± 1.2	ND	ND	80.3 ± 34.5	19.0 ± 8.2
*Artocarpus champeden*	Leaf	44.8 ± 21.3	80.0 ± 27.8	7.6 ± 1.6	526.6 ± 115.7	9.4 ± 3.1
*Garcinia mangostana*	Rind	196.1 ± 65.5	ND	5.0 ± 1.5	282.2 ± 111.2	13.8 ± 7.2
*Nephelium lappaceum*	Rind	333.3 ± 192.5	ND	11.0 ± 8.2	460.6 ± 271.4	23.4 ± 15.7
*Nephelium lappaceum*	Leaf	322.6 ± 1.2	ND	6.3 ± 4.2	261.2 ± 91.7	9.2 ± 5.3
*Peltophorum pterocarpum*	Leaf	1,666.7 ± 986.1	55.9 ± 22.3	6.9 ± 1.7	333.2 ± 121.0	10.0 ± 4.3
*Peltophorum pterocarpum*	Bark	344.8 ± 226.4	370.4 ± 232.8	7.7 ± 2.5	306.8 ± 113.5	0.6 ± 0.04
*Peltophorum pterocarpum*	Pod	ND *	ND	ND	62.5 ± 10.1	1.4 ± 0.9
*Peltophorum pterocarpum*	Flower	434.8 ± 178.2	ND	6.7 ± 2.3	273.6 ± 173.1	10.2 ± 8.4
*Syzygium aqueum*	Leaf	370.4 ± 199.5	5.5 ± 2.8	3.4 ± 1.4	207.2 ± 96.5	4.8 ± 1.7
*Syzygium cumini*	Leaf	232.6 ± 191.3	ND	9.6 ± 2.5	301.6 ± 287.2	11.6 ± 9.7
*Syzygium cumini*	Bark	32.6 ± 11.1	ND	1.2 ± 0.5	152.7 ± 35.6	
*Syzygium cumini*	Seed	93.5 ± 23.5	ND	6.4 ± 1.9	267.4 ± 115.3	
*Vitis vinifera* (Grape)	Seed	256.4 ± 118.9	ND	2.9 ± 0.7	371.0 ± 224.5	
Acarbose		0.286 ± 0.03	83.3 ± 24.5			

* ND, Not detected.

### 2.2. Correlation between Total Phenolic Content, Antioxidant and Antihyperglycemic Activity

**Figure 1:**
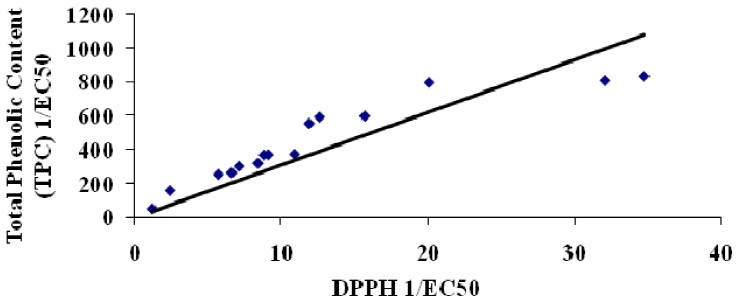
Correlation between Total Phenolic Content and DPPH assay (antioxidant). Correlation = 0.9218.

**Figure 2:**
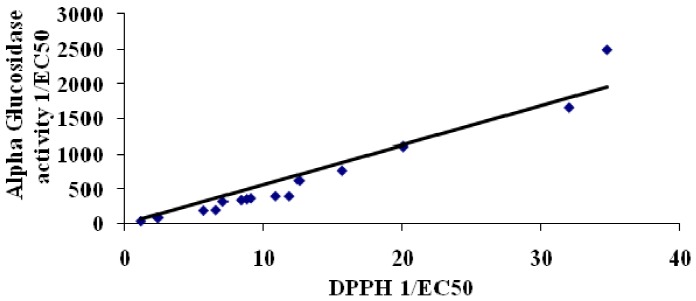
Correlation between α-glucosidase assay (antihyperglycemic) and DPPH assay (antioxidant). Correlation = 0.9734.

**Figure 3:**
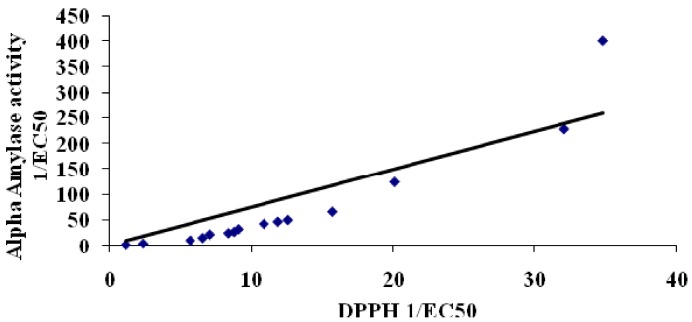
Correlation between α-amylase assay (antihyperglycemic) and DPPH assay (antioxidant). Correlation = 0.9469.

Correlations between total phenolic content and antioxidant activity of plant extracts have been established before [12,13]. In this study, we also established that the total phenolic content of the plant extracts displayed a highly significant correlation (R^2^ = 0.9218) with antioxidant ability (DPPH free radical scavenging activity, Figure 1). A similar study by Ling and coworkers [14] reported that selected Malaysian plant extracts displayed strong correlations between antioxidant ability and total phenolic content. In addition, the antihyperglycemic activity; both α-glucosidase (Figure 2) and α-amylase (Figure 3), also displayed a very strong correlation to antioxidant ability.

Silva Pinto and co-workers [15], have also shown a similar correlation of α-glucosidase activity and antioxidant in *Gingko bilibo* L. leaves extract. This is the first time a correlation between α-amylase activity and antioxidant ability is described.

### 2.3. Tannins Interference in Antihyperglycemic Activity

**Figure 4:**
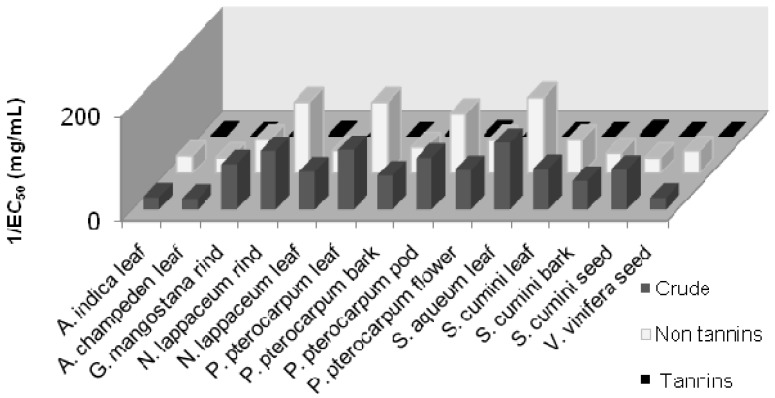
α-Glucosidase inhibition activity in the crude, non-tannin, and tannin extracts of various plants.

**Figure 5:**
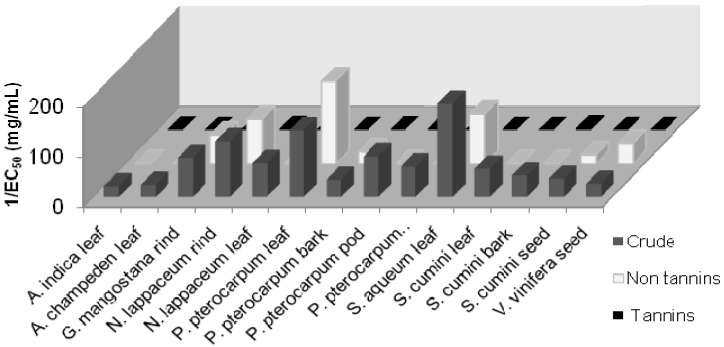
α-Amylase inhibition activity in the crude, non-tannin, and tannin extracts of various plants.

In our efforts to ensure that the α-glucosidase and α-amylase activity in the plant extracts are not due to non-specific absorption of tannins present in the extract, we removed the tannins present in the ethanolic extracts and re-assayed for its α-glucosidase and α-amylase inhibition activities. Figure 4 and Figure 5 show that the tannin extracts do not have any α-glucosidase and α-amylase inhibition activity while both the crude and non-tannin extracts still display significant α-glucosidase and α-amylase inhibition activity.

These results clearly indicate that the antihyperglycemic activity seen in the plant extracts is not due to the tannins.

## 3. Experimental

### 3.1. Plant Collection and Extraction

The 14 local plant parts (rind, bark, leaf, seed, pod, and flower) were obtained from different places in Malaysia. The plants were authenticated by the Herbarium of the Forest Research Institute of Malaysia (FRIM). The plant extracts were standardized using a HPLC method. The method used to prepare the extracts was effectively the same as previously described by [2].

### 3.2. α-Glucosidase and α-Amylase Inhibition Assays

The α-glucosidase and α-amylase inhibition assays was performed using the modified method of [16]. Pancreatic α-amylase (porcine based) and intestinal α-glucosidase (from *Saccharomyces cerevisiae*) were the enzymes used in this study. These enzymes are mainly involves in the digestion of carbohydrate. Inhibition of these enzymes will slower down absorption of glucose into blood thus will reduce postprandial hyperglycemia. Inhibition activity of these enzymes was performed using α-amylase and α-glucosidase colorimetric assays [2]. The absorbance of the inhibition activity of these enzymes was measured using microplate reader-end point reading (BioRad) at appropriate wavelengths; 400 nm (α-glucosidase) and 540 nm (α-amylase). The percentage inhibition was calculated as follows:



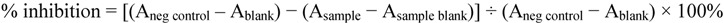



where A = absorbance.

The activity of the plant extracts were assessed by plotting percentage inhibition against a range of plant extracts concentrations. EC_50_ value (effective concentration with 50% inhibition) was thus determined and expressed as means ± SEM of the triplicate measurements.

### 3.3. Antioxidant Assay (DPPH Scavenging Activity)

The DPPH free radicals scavenging activity was assessed according to the modified method of [17]. The percentage inhibition and EC_50_ value was determined as described above.

### 3.4. Determination of Total Phenolic Content (TPC)

The total phenolic content was determined by using the Folin-Ciocalteu method modified according to [18]. The percentage inhibition and EC_50_ value was determined as described above.The content of phenolic compounds in a respective sample was expressed in mg/g of extract, Gallic acid equivalent (GAE).

### 3.5. Precipitation of Tannins by Hide Powder

The method used to remove tannins from plant extracts are based on the adsorption of these compounds onto proteins [19]. Hide powder is used in this study, where 0.06 g is added into 2 mL of crude extract and stirred for 2 h. The tannin adsorbed hide powder is filtered using a membrane funnel (0.02 µm) and filtrate evaporated to evaluate its concentration, the solution is a tannin-free ethanol extract.

## 4. Conclusions

It was concluded that ethanolic extracts of a variety of Malaysian plant parts displayed far better antihyperglycemic (α-glucosidase and α-amylase), antioxidant (DPPH scavenging ability) and total phenolic content than the corresponding aqueous extracts. Among the 14 plant extracts tested, *Peltophorum pterocarpum* (bark and flower parts) exhibited extremely high α-glucosidase, α-amylase and antioxidant, as well as total phenolic content. The antihyperglycemic activity of this plant extract was seen to be far better than that of the antidiabetic drug acarbose. In addition, there was a strong correlation between the antihyperglycemic (α-glucosidase and α-amylase inhibition activity) and antioxidant ability. This is the first study showing a correlation between α-amylase inhibition activity and antioxidant ability of these plant extracts. Finally, we confirmed that the antihyperglycemic activity of the plant extracts was not due to the non-specific absorption of tannins.
